# Single‐arm interventional versus observational studies for assessing efficacy: A meta‐epidemiological study

**DOI:** 10.1002/cesm.70016

**Published:** 2025-01-16

**Authors:** Mary Chappell, Deborah Watkins, Alice Sanderson, Lavinia Ferrante di Ruffano, Paul Miller, Hariet Fewster, Anita Fitzgerald, Mary Edwards, Rachael McCool

**Affiliations:** ^1^ York Health Economics Consortium, Innovation Way University of York York UK

**Keywords:** bias, clinical trial, observational study, treatment outcome

## Abstract

**Introduction:**

Interventional single‐arm trials (SATs) are increasingly being used as evidence, despite a lack of agreement on their validity and where they should sit in the hierarchy of evidence. We conducted a meta‐epidemiological study to investigate whether there are systematic differences in outcomes and levels of between‐study heterogeneity for SATs compared with their observational counterpart, single‐arm cohort studies.

**Methods:**

We identified systematic reviews (SRs) of pharmacological interventions, published in 2023, that included both interventional and observational single‐arm studies. For each SR, subgroup meta‐analysis of dichotomous outcomes was conducted for included SATs and single‐arm cohort studies to assess effect sizes, levels of heterogeneity and between group differences. In a sensitivity analysis, clinically heterogeneous primary studies were removed and analyses re‐run.

**Results:**

66 SRs contained single‐arm studies, of which 13 reported meta‐analyses of dichotomous efficacy outcomes. There was no overall risk difference for SATs compared with single‐arm cohort studies (risk difference: −0.020, 95% CI: −0.092 to 0.052, *p* = 0.59). In the sensitivity analysis, there was a tendency to higher effect for single‐arm cohort studies, but no significant difference (risk difference: −0.071, 95% CI: −0.161, 0.019, *p* = 0.12). There were high levels of between‐study heterogeneity within both SATs (median; range *I* ^2^: 54.8; 11.3–91.0) and single‐arm cohorts (median; range *I* ^2^: 77.2; 0–94.7) and heterogeneity remained high in the sensitivity analysis.

**Conclusion:**

There do not appear to be systematic differences in outcome between SATs and single‐arm cohort studies, but further research is recommended to confirm this finding. Levels of heterogeneity are high within both designs, even after attempts to reduce clinical heterogeneity. Because clinical heterogeneity had potentially been removed, remaining statistical heterogeneity may have been due to bias related to study conduct. Future work should utilize larger samples and additional methods to further clarify the relative validity of single‐arm designs.

## 1 BACKGROUND

The randomized controlled trial (RCT) is the gold standard design for estimating the effectiveness of interventions and is required by most health technology assessment (HTA) agencies for evidence of effectiveness and safety. However, approvals are increasingly being granted on the basis of single‐arm trials (SATs) [1, 2, 3, 4, 5], where interventions are either tested in single group of patients without reference to a control group, or an externally‐derived control group is used.

For SATs, it is argued that findings can be misleading due to selection and measurement bias [6], and could be an inefficient approach when an RCT is subsequently likely to be required [7]. In some countries, new guidance points to the avoidance of SATs, and a requirement for RCTs to demonstrate evidence of efficacy and safety [8]. Other guidance may limit the use of SATs as the sole evidence‐base for applications [6]. However, in some disease areas, RCTs are deemed unethical, and SATs continue to be the primary source of evidence.

Within the accepted “hierarchy of evidence,” there is no existing guidance on where SATs should be positioned. Published evidence hierarchies [9, 10] do not include SATs or what may be considered their closest counterpart, observational single‐arm studies of the effect of interventions or exposures (single‐arm cohort studies). However, given the increasing number of SATs conducted, and the, sometimes, congenial receipt of SATs in HTA submissions, the assumption appears to be that SATs provide higher quality evidence compared to single‐arm cohort studies.

We conducted a meta‐epidemiological study to investigate the validity of SATs compared with single‐arm cohort studies by evaluating their relative effect sizes and consistency of findings in studies of pharmacological interventions.

## 2 METHODS

### 2.1 Approach

To achieve our aims, we planned to identify a recent sample of systematic reviews of pharmacological interventions that included both interventional and observational single‐arm studies. We developed a protocol for internal use (Supplementary Material S1). It was not registered, but contained the eligibility criteria, information sources, search methods and methods and items for data collection. Since this work also involved data collection to answer two other research questions, the protocol covered details of methods for all questions.

To explore the relative validity of SATs and single‐arm cohort studies we:

1) Compared the difference in effect size of SATs with single‐arm cohorts within each review and across reviews. The aim of this was to identify any systematic difference in outcome between study designs. In a sensitivity analysis, we removed clinically heterogenous studies, with the aim of identifying any residual differences that could potentially be attributed to bias due to study conduct.
2) Compared levels of heterogeneity within groups of SATs and single‐arm cohorts for each review. Following removal of clinically heterogeneous studies in a sensitivity analysis, we aimed to identify any residual heterogeneity that could potentially be attributed to bias due to study conduct.

### 2.2 Searches and eligibility criteria

We conducted a highly pragmatic literature search to create a sample of recent systematic reviews that included single‐arm studies (Supplementary Material S1). The sample was restricted to systematic reviews of pharmacological interventions. Searches were conducted in MEDLINE and the Cochrane Database of Systematic Reviews (CDSR) for reviews published from January to July 2023. The search strategy was devised using a pragmatic selection of subject indexing terms and free text search terms in the Title, Abstract and Keyword Heading Word fields. Terms for three concepts were combined as follows: single‐arm studies AND pharmacological interventions AND a systematic review filter. The study design filter was based on a published filter devised by CADTH [11]. The strategy excluded animal studies and some publication types (editorials, news items and case reports) and was restricted to studies published in English.

Systematic reviews were included if they had a defined research question, searched two or more databases, reported a list of included studies and reported one or more efficacy outcomes. Eligible systematic reviews contained single‐arm trials and single‐arm cohorts and may have also included comparative studies. However, reviews of only comparative studies were excluded.

### 2.3 Study selection and data extraction

A single reviewer identified eligible systematic reviews and determined the number of SATs and single‐arm cohorts included in each review. SATs were defined as prospective interventional single‐arm studies involving treatment outside of routine practice. Single‐arm cohorts were defined as observational single‐arm studies and could include a wide range of designs, including retrospective chart reviews and prospective registry studies. Where review authors appeared to have misclassified single‐arm studies, we recorded the nature of misclassification.

Information on the characteristics of each systematic review were extracted by a single reviewer. For the primary studies in each review, data for the primary binary outcome were extracted by a single reviewer and checked by a second reviewer. Where reviews reported a number of interventions and/or primary outcomes, the intervention or outcome with the largest number of contributing studies was selected for extraction.

### 2.4 Analysis

For each included review, sub‐group analysis was conducted on dichotomous outcome data for SATs and single‐arm cohorts in OpenMeta [12] using DerSimonian‐Laird random effects meta‐analysis. The outcomes of these meta‐analyses were compared within each review using the difference between effect sizes for SATs and single‐arm cohorts (risk difference) and the corresponding standard errors (SE). The risk difference and corresponding SEs for each review were meta‐analysed using generic inverse variance random effects meta‐analysis.

The risk ratio was not computed because, in a number of cases, review authors did not report raw data (events and sample size), but only reported effect size with 95% confidence intervals (CIs). In these cases, it appeared that CIs had been calculated without log transformation so the standard errors on the log scale could not be extracted. It was therefore not possible to reliably calculate the SE for a log risk ratio.

Statistical heterogeneity was quantified used *I* ^2^, where substantial heterogeneity was considered to be *I* ^2^ ≥ 50%. Statistical heterogeneity for SATs and single‐arm cohorts within each review was summarized with median and range and the Wilcoxon signed‐rank test was used to compare heterogeneity between paired samples of SATs and single‐arm cohorts across reviews. Due to the limited data, the Mann Whitney test was also used to compare unpaired samples of SATs and single‐arm cohorts across reviews.

For the sensitivity analysis, studies considered to be clinically heterogeneous were removed. Blind to the results of primary studies and reviews, two reviewers assessed the study characteristics tables of each review and reached a consensus on the selection of studies that were considered clinically heterogeneous. These studies were removed, and all analyses were re‐run.

## 3 RESULTS

### 3.1 Results of searches

The search retrieved 903 unique records. Following title/abstract and full‐text screening, we identified 66 eligible systematic reviews of pharmacological interventions. Of these, 13 reported binary outcome data for SATs and single‐arm cohorts in meta‐analyses and were included for investigation of outcome data.

### 3.2 Included reviews

Characteristics of the 13 reviews are shown in Table 1. 9 of 13 reviews evaluated pharmacological interventions in patients with cancer [15, 16, 17, 20, 21, 22, 23, 24, 25]. Other reviews assessed interventions in jaw osteoradionecrosis [13], migraine [14], depressive disorder [18] and pustulosis [19]. Eight reviews had registered a protocol on PROSPERO [14, 15, 16, 17, 18, 20, 22, 24]. No reviews reported funding from a pharmaceutical company. Each review contributed between 1 and 33 SATs and between 1 and 18 single‐arm cohorts to the meta‐analysis.

**Table 1:** Characteristics of systematic reviews included in the analysis.

	Author, year	Prospero no.	Funding source	Condition	Intervention	Outcome*	*N* included SATs$	*N* included single‐arm cohorts$
1.	Arqueros‐Lemus 2023 [13]	NR	Indian Council of Medical Research (ICMR)	Osteoradionecrosis of the jaws	Pentoxifylline with tocopherol	Healthy (defined as full mucosal coverage without exposed bone)	2	1
2.	Bomtempo 2022 [14]	CRD42022335137	NR	Migraine	Erenumab	≥50% reduction in monthly migraine days	7	7
3.	Gao 2023 [15]	CRD42022377004	Natural Science Foundation of Sichuan	Advanced hepatocellular carcinoma	Atezolizumab plus bevacizumab	Objective response rate	1	15
4.	Guo 2023 [16]	CRD42022335072	None	Malignant pleural mesothelioma	Chemotherapy	Objective response rate	5	7
5.	Li 2023a [17]	CRD42023390316	NR	HER2‐low/positive advanced breast cancer	Trastuzumab deruxtecan	Objective response rate	7	1
6.	Li 2023b [18]	CRD42022362161	National University of Ireland, Galway	Major depressive disorder	Vortioxetine	Remission rate	1	8
7.	Spencer 2023 [19]	NR	None	Palmoplantar psoriasis and palmoplantar pustulosis	Apremilast	PPPASI 75	1	2
8.	van Weelden 2023 [20]	CRD42018089801	Key Projects of Philosophy and Social Sciences Research	Advanced and recurrent endometrial cancer	Progestin	Overall response rate	2	18
9.	Wei 2023 [21]	NR	None	Early‐stage endometrial cancer	Levonorgestrel‐releasing intrauterine system‐based therapies	Complete response	2	13
10.	Wu 2023 [22]	CRD42022331456	Hebei Clinical Research Center for Radiation Oncology	Nonsmall cell lung cancer	Immunotherapy	Pathologic complete response	33	16
11.	Xian 2023 [23]	NR	NR	Solid tumors	PD1/PDL1 inhibitors	Response rate	2	4
12.	Zeng 2023 [24]	CRD42023396057	NR	Glioma	PD‐1/PD‐L1 inhibitors	1 year progression free survival	9	1
13.	Zhou 2023 [25]	NR	None	Inflammatory Bowel Disease	Tyrosine Kinase Inhibitors	Partial response	3	2

* Outcome selected for meta‐analysis in this investigation.

^$^ Number of SATs and single‐arm cohorts included in meta‐analysis for selected outcome.

### 3.3 Outcomes

#### 3.3.1 Effect size

**Figure 1:**
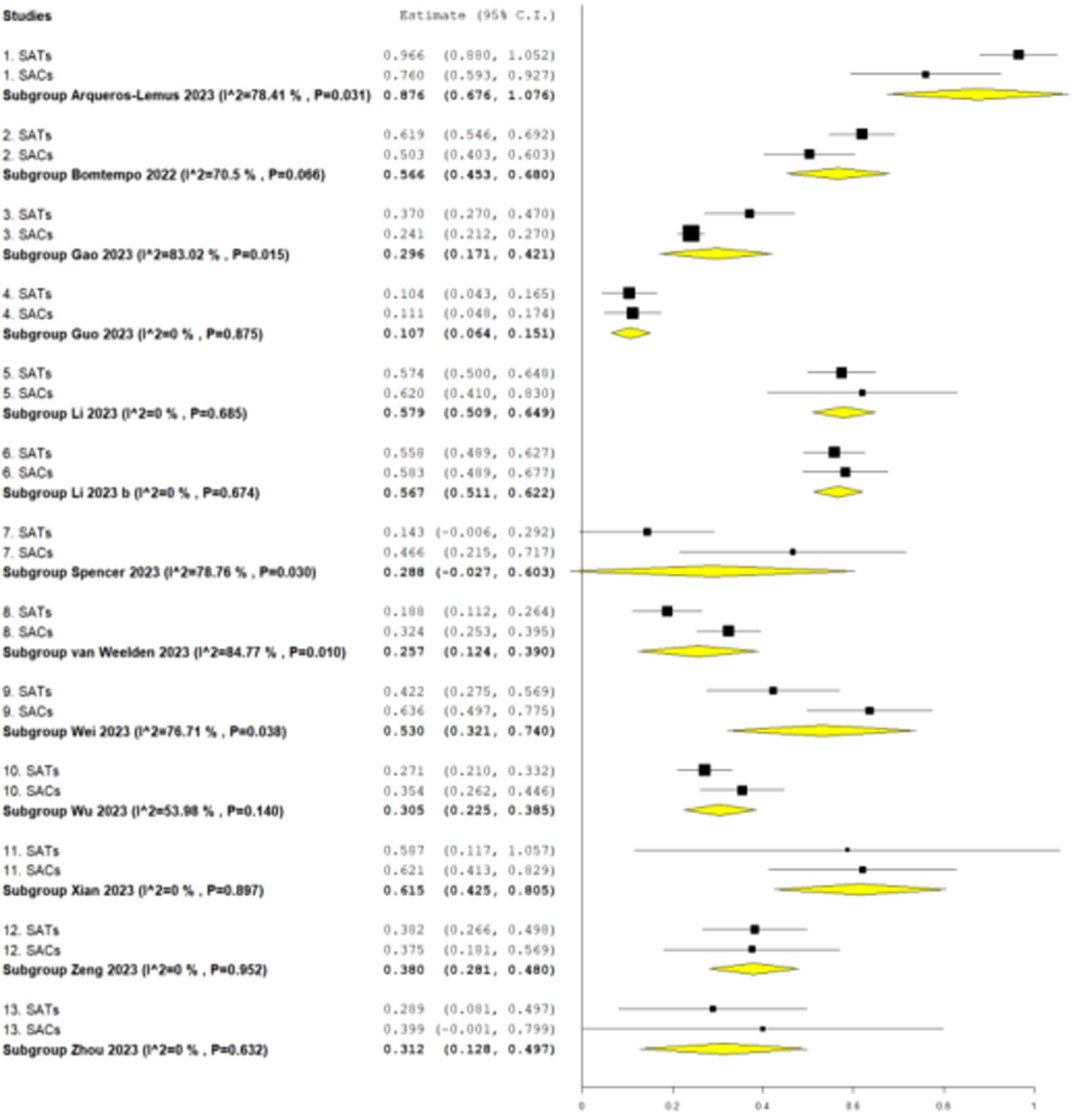
Effect estimates (proportions) for binary outcomes measured in SATs and in single‐arm cohorts (SACs) in the 13 included reviews. For each review (numbered 1 to 13), the pooled effect estimate for SAT and single‐arm cohort groups is presented.

**Figure 2:**
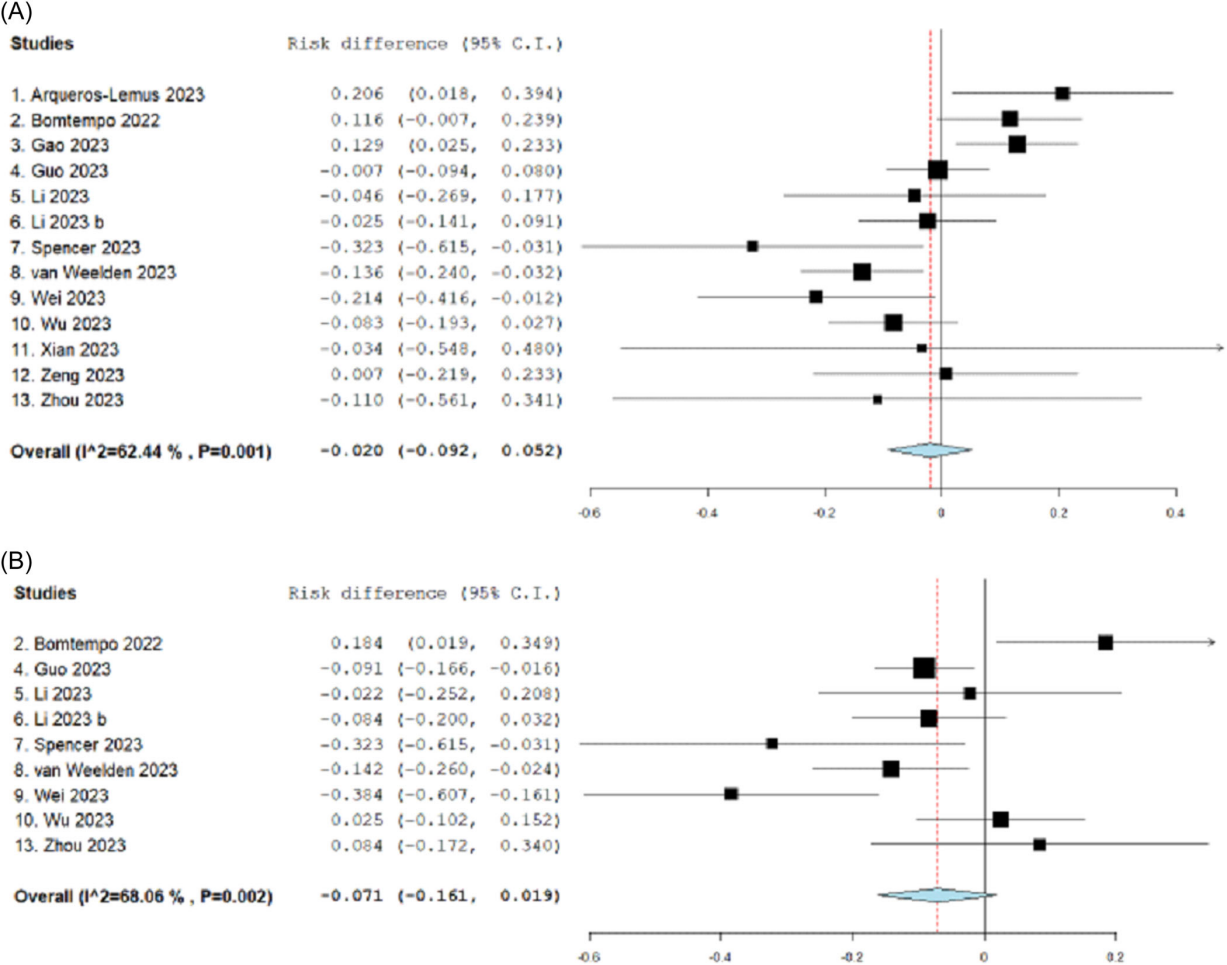
Risk difference for SATs versus single‐arm cohorts across (A) all included reviews, (B) reviews in the sensitivity analysis (positive risk difference indicates a higher estimate for SATs, negative risk difference indicates higher estimate for single‐arm cohorts).

Effect sizes and variability for each within‐review SAT and single‐arm cohort meta‐analysis are shown in Figure 1. Results were mixed: single‐arm cohorts produced significantly higher effect estimates (proportions) compared to SATs in three reviews (reviews 7, 8 and 9 in Figure 1) [19, 20, 21], significantly lower effect estimates in two reviews (reviews 1 and 3 in Figure 1) [13, 15], and were not significantly different to SATs in eight reviews [14, 16, 17, 18, 22, 23, 24, 25]. In terms of ranking, nine reviews [14, 16, 17, 18, 19, 20, 21, 22, 23, 25] yielded higher estimates in meta‐analyses of single‐arm cohorts and four reviews [13, 14, 15, 24] had higher effect estimates for SATs. Overall, there was no difference in the computed risk difference across reviews for SATs compared with single‐arm cohorts (Figure 2A) (risk difference: −0.020, 95% CI: −0.092 to 0.052, *p* = 0.59).

**Figure 3:**
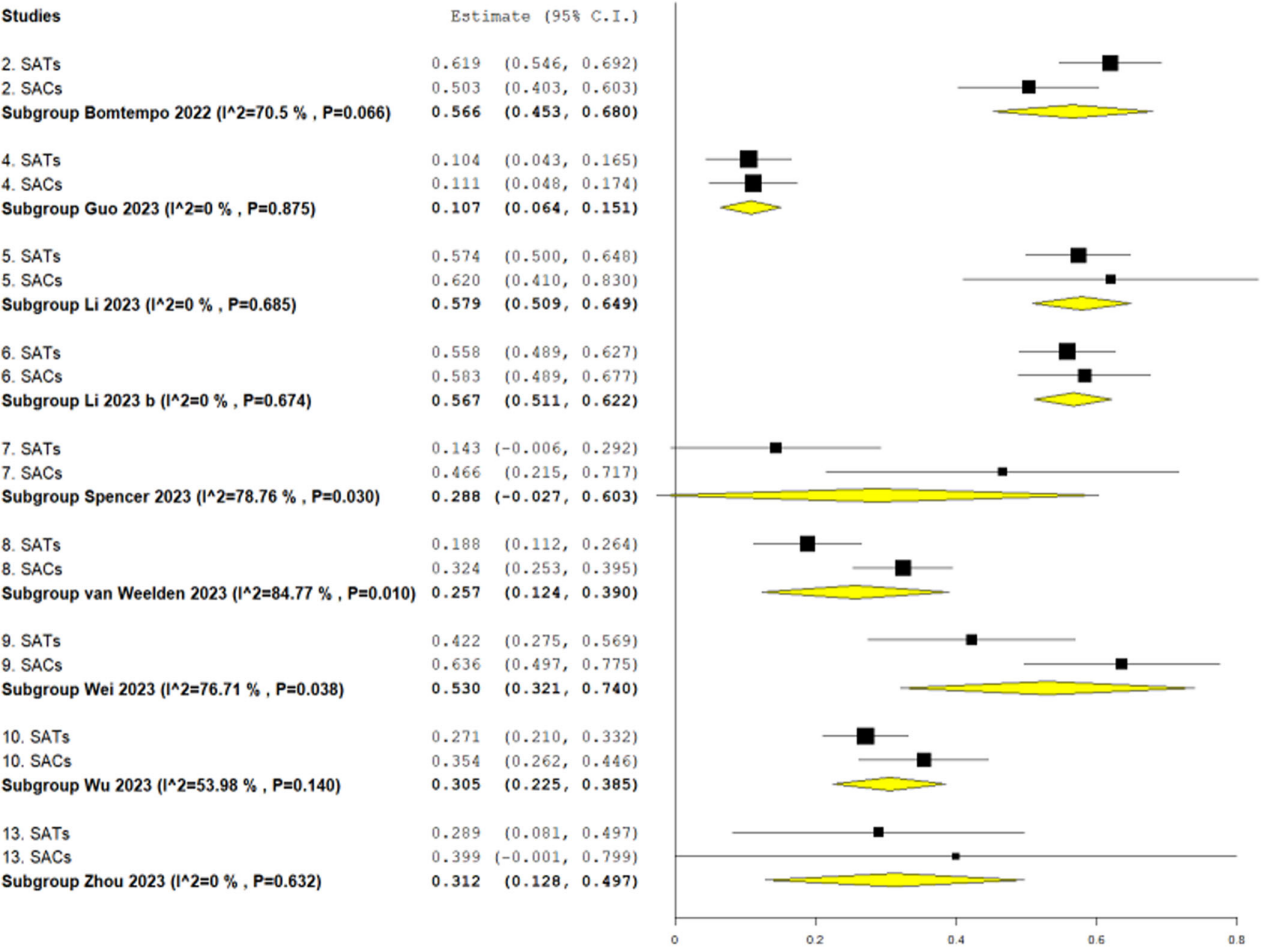
Effect estimates (proportions) for binary outcomes measured in SATs and in single‐arm cohorts (SACs) in the 11 reviews retained in the sensitivity analysis. For each review (numbered 1–13), the pooled effect estimate for SAT and single‐arm cohort groups is presented.

In the sensitivity analysis, where clinically heterogeneous primary studies were removed from meta‐analyses, there were similar findings (Figure 3). Four reviews dropped out of the analyses because there were no remaining SATs or single‐arm cohorts. Of the nine reviews with remaining paired data, four produced significantly higher pooled effect estimates for single‐arm cohorts [16, 19, 20, 21] (reviews 4, 7, 8 and 9 in Figure 3), one produced a significantly higher pooled effect estimate for SATs [14] (review 2 in Figure 3, and there was no significant difference in the remaining four reviews [17, 18, 22, 25]. In terms of ranking, six reviews yielded higher estimates for single‐arm cohorts [16, 17, 18, 19, 20, 21] and three reviews produced higher effect estimates for SATs [14, 22, 25]. In the overall risk difference comparison, there was a tendency to greater effect for single‐arm cohorts compared with SATs across the nine reviews but no significant difference (Figure 2B) (risk difference: −0.071, 95% CI: −0.161, 0.019, *p* = 0.12).

#### 3.3.2 Heterogeneity

In most reviews, heterogeneity was high for both SATs (median *I* ^2^: 54.8%, range: 11.3%–91.0%) and single‐arm cohorts (median *I* ^2^: 77.2%, range 0%–94.7%). There was no significant difference between the two designs in levels of heterogeneity when tested using the Wilcoxon signed‐rank test for paired within‐reviews data (*W* = 10; *Z* = −0.6761, *p* = NS) or the Mann‐Whitney test on unpaired data (*U* = 43, *p* = 0.62414).

**Figure 4:**
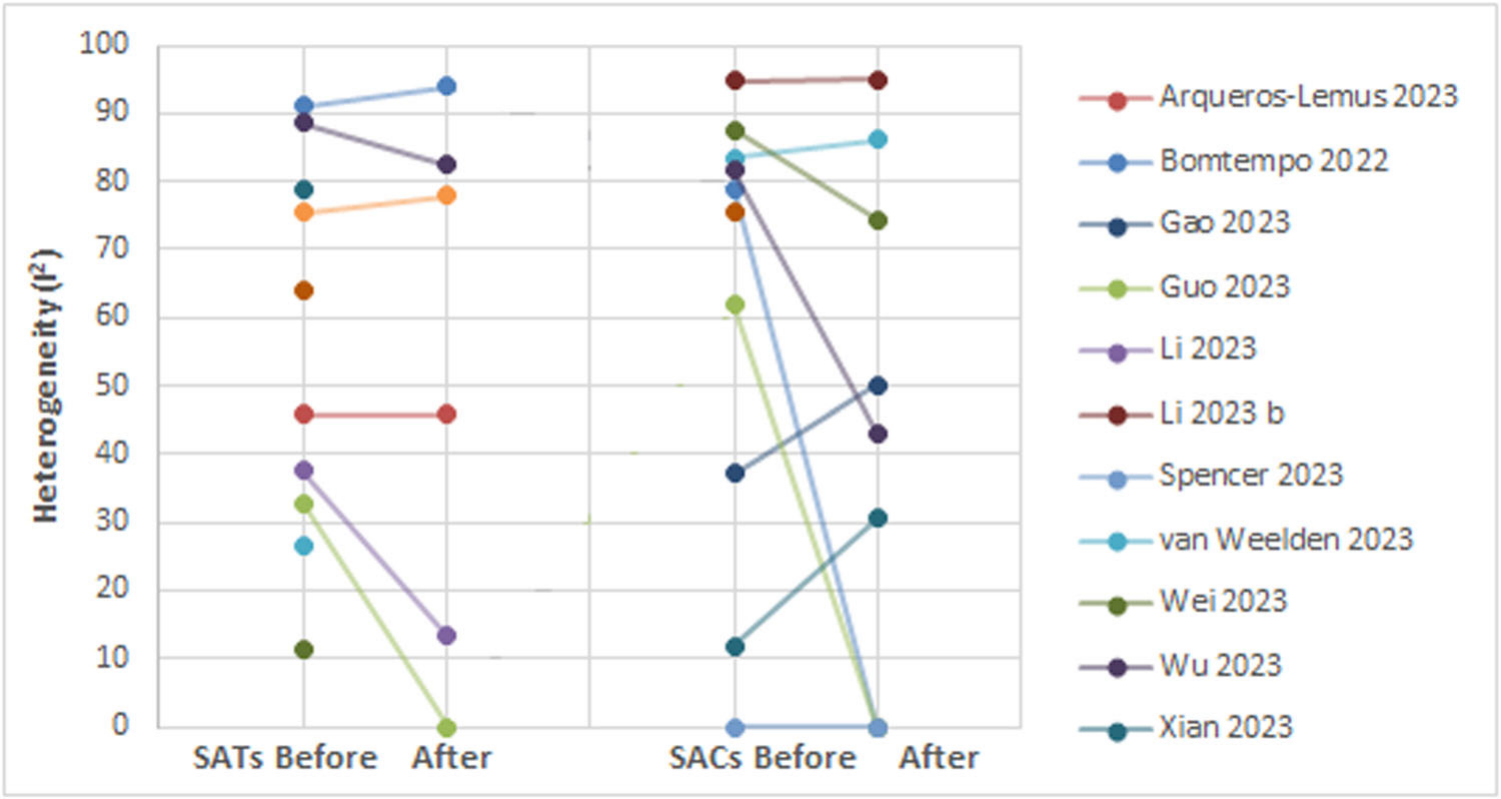
Heterogeneity in reviews including SATs and single‐arm cohorts before and after the removal of clinically heterogenous studies. NB single points represent groups of SATs or single‐arm cohorts with only one study remaining in the sensitivity analysis.

In sensitivity analysis, where clinically heterogeneous studies were removed, there was a tendency to reduced levels of heterogeneity for SATs and single‐arm cohorts (Figure 4). However, levels of heterogeneity remained high (SATS: median *I* ^2^ 61.7%, range: 0%–93.8% single‐arm cohorts: median *I* ^2^ 43.1%, range 0%–95.0%). There was still no significant difference in levels of heterogeneity for SATs versus single‐arm cohorts: Mann‐Whitney *U* = 22.5, *p* = 0.638 (too small sample size to allow reliable calculation with Wilcoxon signed‐rank).

## 4 DISCUSSION

Unlike for comparative studies, where RCTs are considered superior to other comparative designs, the relative value of SATs amongst other single‐arm studies is unclear. Their intent and conduct are different to single‐arm cohort studies, and they therefore have different potential sources of bias. It is difficult to quantify the effect of these biases on outcomes and to make judgements about the relative value of different single‐arm designs. We have endeavored to explore these issues through empirical study of a small sample of systematic reviews.

The findings of this work suggest that there may be no overall systematic difference in results from interventional compared with observational single‐arm studies. The pooled risk difference in dichotomous outcomes for SATs versus single‐arm cohorts across reviews was small and insignificant (0.020, 95% CI: −0.092 to 0.052), but there was significant heterogeneity (*I* ^2^: 62.44%, *p* = .001). This is aligned with previous meta‐epidemiological research [26, 27]. Toews et al. found that, in a sample of systematic reviews comparing outcome for RCTs with comparative observational studies, there was no differences in the relative outcome across reviews [26, 27].

However, in our study, and the study by Toews et al., there appeared to be a number of within‐review differences pointing to “something going on.” We found significantly higher effect sizes for single‐arm cohort studies in three reviews (23%) and for SATs in two reviews (15%), with no significant difference in the other eight reviews. Toews et al. observed similar proportions with significant differences: 21% of reviews had significantly higher effect sizes for RCTs and 12% had significantly higher effect sizes for comparative observational studies.

It may be argued that heterogeneity in study and population characteristics could introduce these differences in findings for different study designs within reviews. However, in our work, when clinically heterogeneous studies were removed, significant differences in outcome for single‐arm cohorts versus SATs remained. This indicates the possibility that differences in study conduct or bias were responsible for within review differences in effect size.

There was also an indication of the potential influence of bias in investigations of heterogeneity. Heterogeneity within groups of SATs and single‐arm cohorts was high. Following the removal of clinically heterogeneous studies, there was a decrease in heterogeneity in some but not all reviews and, in some cases, for both SATs and single‐arm cohorts, heterogeneity remained high or even increased. Since clinical heterogeneity was accounted for, the remaining statistical heterogeneity may be due to differences in study conduct, including bias.

Research on bias in single‐arm study designs points to measurement (including assessment) and selection bias (including bias from attrition) as critical [6, 28]. Measurement bias can be introduced by the mode of measurement and/or assessment. The utilization of inaccurate measures that are likely to systematically under‐ or over‐estimate treatment effects introduces bias. This may be a common problem in retrospective observational evidence, such as retrospective single‐arm cohorts, where investigators must make do with whatever outcome measures are available (in pre‐planned SATs and prospective single‐arm cohorts, more accurate outcome measures can be planned). A systematic under‐ or over‐estimation of a measurement may lead to inflation or deflation of the reported effect. Where there is variation in this bias across single‐arm cohort studies, causing over‐estimation in some studies and under‐estimation in other studies, this results in increased observed statistical heterogeneity.

Measurement bias can also be introduced where outcome assessors are not independent from the study team. Where outcomes are measured by study personnel, there may be bias in their assessment and reporting, potentially inflating estimations of treatment effect. This may be anticipated to potentially be more common in prospective studies, particularly in SATS that are industry funded and outcome assessors are part of the industry personnel team. However, retrospective single‐arm cohorts can also be subject to measurement bias if personnel extracting information from historic records are not impartial. Where there is variation across studies in the extent of this bias, we may anticipate higher levels of statistical heterogeneity. It could potentially be anticipated that statistical heterogeneity in SATs is not influenced by this type of measurement bias where it operates in most trials. But heterogeneity from this type of measurement bias could be introduced by variation in the independence of outcome assessors in single‐arm cohorts.

Although measurement bias may affect both SATs and single‐arm cohorts, selection bias is likely to be a more extensive problem in single‐arm cohorts. A common feature of observational evidence is that data is only reported for patients for whom there is complete outcome data, introducing bias into effect estimates. This scenario is less likely with SATs, where more rigorous procedures for patient inclusion and follow‐up are likely to be in place. However, SATs may be more likely to suffer from poor generalizability of the patient population, with inclusion criteria designed to result in higher effect sizes. Related to this is regression to the mean, introduced when patients are selected for inclusion based on elevated disease severity. SATs may particularly suffer, where stringent inclusion criteria result in high baseline severity that naturally regresses to the mean on follow‐up. In terms of observed statistical heterogeneity, SATs may be less affected. We may anticipate effects sizes in SATs to be inflated by selection bias but, if this is a reasonably uniform feature (with common poor generalizability), we may not anticipate increased statistical heterogeneity. However, in single‐arm cohorts, where there may be variation, with some studies only reporting outcome for patients with follow‐up data but other studies reporting the full data set, selection bias may result in increased observed statistical heterogeneity.

In the examined reviews, bias appears likely to have had an impact on the effect size and observed heterogeneity. The implication is that risk of bias assessment is important to identify and consider the impact of key elements of bias. Tools should be used that consider domains relevant to different study designs. There are a number of tools developed for the evaluation of single‐arm cohort studies, although these are often referred to as case series tools [28, 29, 30]. Amongst other elements, these tools contain domains relating to the inclusion of patients (whether consecutive and complete) and the validity of outcome measures, and may be considered to cover encountered issues reasonably well.

However, the availability of tools for assessing SATs is more limited and further work may be required in their development. The MINORs tool has been designed for the assessment of SATs and comparative nonrandomised trials [31]. It contains items related to the unbiased evaluation of outcomes and appropriateness of the length of follow‐up. However, the suitability of eligibility criteria is not examined, with no items related to restrictions placed on demographic or clinical characteristics. Reviewers may be expected to highlight these areas in consideration of external validity, but they may also be important in the assessment of internal validity/risk of bias. For example, regression to the mean may be an important moderator of effect size. Some SATs recruit people on the basis of elevated baseline measurements and, although these measurements may have reasonably good generalizability to populations with the condition, the impact on estimated effect size needs to be considered. Another tool available for the assessment of SATs is provided by the US National Heart, Lung and Blood institute (Tool for Before‐After (Pre‐Post) Studies With No Control Group) [30]. This tool does incorporate criteria related to enrollment onto the study, as well as items for suitability of outcome measures and blinding of outcome assessors and may be a suitable option.

However, there may be cases where reviewers wish to use a tool designed for the assessment of single‐arm cohorts (often termed ‘case series’) for SATs e.g. where the review contains both and reviewers wish to use only one tool. In these cases, it is important to consider SAT‐specific scenarios for the assessment of SATs. For example, for the assessment of measurement bias, not only the methods used but the independence of the person conducting outcome assessment, should be judged. Reference to a SATs tool, for example the National Heart, Lung and Blood institute tool, would help to identify additional prompts. These additional considerations should be made clear in the methods and reporting and feed into the overall risk of bias assessment.

This review is limited by the small number of included reviews. A larger sample may have allowed more conclusive findings. A number of observations were noted, including, in the sensitivity analysis, the tendency for single‐arm cohorts to demonstrate greater effect sizes compared with SATs (Figure 3), and the lack of reduction in heterogeneity (Figure 4). Other authors have observed a tendency to larger effect sizes in nonrandomised compared with randomized comparative studies [32]. Since the majority of nonrandomised studies they considered were observational, this is akin to the observational versus interventional comparison in our work. A greater sample size might have enabled us to come to firm conclusions on the nature of these observations.

Another potential limitation was in the analysis, where we computed risk difference rather than risk ratio across reviews. This was necessary because raw event data was not available for all reviews and it was not possible to obtain log standard errors where only effect sizes and CIs were presented. Other authors have used ratios to compare effect sizes across reviews [27]. This may give greater adjustment for between‐review heterogeneity and be the preferred option. However, computing a risk difference allows us to observe general patterns in the data and we believe that it is helpful to present the data in this way.

A final limitation may be in the sensitivity analysis, where we based judgements on clinical heterogeneity from information reported in review study characteristics tables. Where data was missing, or where there were unknown confounders, we may have missed important sources of clinical heterogeneity. However, although we were unlikely to have identified all clinically heterogenous studies, there was consistency in opinion between reviewers, and we believe that clinical heterogeneity was reduced. The lack of a reduction in statistical heterogeneity therefore appears to point to the presence of bias.

Further research is important to investigate the value of different types single‐arm evidence. Larger sample sizes are important as well as alternative methods to corroborate findings. Since effect size alone cannot be used as an indicator of bias, nonclinical heterogeneity may be key, reflecting variability that cannot be explained by differences in clinical characteristics. Further research could involve extraction of risk of bias assessments and include investigation of specific elements of bias, such as measurement and selection biases, to compare the extent to which they are present in SATs and single‐arm cohorts and investigate how their presence impacts outcomes.

## 5 CONCLUSION

There do not appear to be systematic differences in effect sizes between SATs and single‐arm cohort studies that evaluate pharmaceutical treatments. However, in clinically homogeneous samples of studies, single‐arm cohort studies may yield larger effects. Heterogeneity in effect size was high within both designs, even after attempts to reduce clinical heterogeneity, indicating that bias related to study conduct may have an impact on outcomes. Future work should utilize larger samples and additional methods to further clarify the relative validity of single‐arm designs.

## AUTHOR CONTRIBUTIONS

Mary Chappell collected, analysed and interpreted the data and drafted the manuscript. Deborah Watkins collected and analysed the data and reviewed the manuscript. Alice Sanderson collected the data and reviewed the manuscript. LF advised on project methods and reviewed the manuscript. Anita Fitzgerald collected the data and reviewed the manuscript. Paul Miller designed and conducted searches and reviewed the manuscript. Hariet Fewster advised on statistical analysis and reviewed the manuscript. Mary Edwards advised on project methods and collected data. Rachael McCool advised on project methods and reviewed the manuscript.

## CONFLICT OF INTEREST STATEMENT

The authors declare no conflicts of interest.

## Supporting information

Supporting information.

Supporting information.

## Data Availability

The datasets analysed during the current study are available from the corresponding author on reasonable request.
