# Supplementary material for: Single‐arm interventional versus observational studies for assessing efficacy: A meta‐epidemiological study
Source: Cochrane Evid Synth Methods. 2025 Jan 16;3(1):e70016. doi: 10.1002/cesm.70016 (PMC11795979; doi:10.1002/cesm.70016)
Supplement: Supplementary file 1 — Supporting information. [file CESM-3-e70016-s002.docx]

Single-arm studies methods work

Protocol v0.1

**MARY CHAPPELL, Senior Research Consultant**

MARY EDWARDS, Senior Research Consultant

LAVINIA FERRANTE DI RUFFANO, Senior Research Consultant

ANITA FITZGERALD, Senior Research Consultant

PAUL MILLER, Information Specialist

**DEBORAH WATKINS, Research Consultant**

**ALICE SANDERSON, Research Consultant**

18/07/2023

Table of Contents

[1 Background 3](#_Toc140588223)

[2 Eligibility Criteria 4](#_Toc140588224)

[2.1 Population 4](#_Toc140588225)

[2.2 Interventions and Comparators 4](#_Toc140588226)

[2.3 Outcomes 4](#_Toc140588227)

[2.4 Study Design 4](#_Toc140588228)

[2.5 Limits 4](#_Toc140588229)

[2.6 Protocol Amendments 5](#_Toc140588230)

[3 Methods 6](#_Toc140588231)

[3.1 Identifying Relevant Studies 6](#_Toc140588232)

[3.2 Study Selection 8](#_Toc140588233)

[3.3 Data Extraction 8](#_Toc140588234)

[3.4 Synthesis and Analysis 9](#_Toc140588235)

[4 Timelines 9](#_Toc140588236)

[4.1 Timeline 9](#_Toc140588237)

[5 References 10](#_Toc140588238)

[Appendix A – Protocol Amendments 11](#_Toc140588239)

All reasonable precautions have been taken by YHEC to verify the information contained in this publication. However, the published material is being distributed without warranty of any kind, either expressed or implied. The responsibility for the interpretation and use of the material lies with the reader. In no event shall YHEC be liable for damages arising from its use. York Health Economics Consortium is a Limited Company. Registered in England and Wales No. 4144762. Registered office as shown.

# Background

Single-arm trials are increasingly being used as evidence in regulatory body submissions. A single-arm variety of observational evidence also exists, that can be classed as a ‘single-arm cohort study’, consisting of both large prospectively collected data and small retrospective chart reviews.

To understand the different types of single-arm studies in use and evaluate their usefulness as evidence, we will conduct a review of recently published single-arm studies. We will do this by reviewing recently published systematic reviews that include single-arm studies to answer the following research questions:

- What type of studies are included in reviews of non-comparative studies?
- What risk of bias tools are used for the assessment of single-arm trials and cohort studies?
- Is there a systematic difference in outcome for different study designs?

# Eligibility Criteria

The eligibility criteria are summarised in Table 2.1.

## Population

- Systematic reviews of any population.

## Interventions and Comparators

- Systematic reviews of any pharmacological intervention.

## Outcomes

- Systematic reviews of efficacy or efficacy and safety.
  - Reviews of safety alone would be excluded but tagged.

## Study Design

- Systematic reviews containing single-arm studies (with or without other study designs).

## Limits

- Systematic reviews published in 2023.

Primary studies, narrative or non-systematic reviews, news items, editorials, and preprints of any form will be ineligible. Conference abstracts will be ineligible.

## Protocol Amendments

Any essential protocol amendments or clarifications will be recorded in Appendix A. Changes will be made to the text of the protocol and flagged with [PA#].

# Methods

## Identifying Relevant Studies

### Search strategy

MEDLINE (OvidSP) and the Cochrane database of systematic reviews (CDSR) were searched using title, abstract and indexing terms for:

- Drug/pharmacological therapy.
- Single-arm.

The strategy was devised using a combination of subject indexing terms and free text search terms in the Title, Abstract and Keyword Heading Word fields. The search terms were identified through discussion within the research team, scanning background literature, browsing database thesauri and use of the PubMed PubReminer tool (<http://hgserver2.amc.nl/cgi-bin/miner/miner2.cgi>).

The strategy excludes animal studies from MEDLINE using a standard algorithm. The strategy also excludes some ineligible publication types which are unlikely to yield relevant study reports (editorials, news items and case reports) and records with the phrase 'case report' in the title.

The search is restricted the English language and to publications from 2023 onwards.

Figure 3.1: Search strategy for Ovid MEDLINE® ALL and CDSR

Final search used - single arm - methods project - MEDLINE

Search date: 17 July 2023

Ovid MEDLINE(R) ALL <1946 to July 13, 2023>

1 single arm.ti,ab,kf. 13097

2 chart review?.ti,ab,kf. 53365

3 case series.ti,ab,kf. 103841

4 (uncontrolled adj3 (evidence or study or studies or trial or trials or data)).ti,ab,kf. 6868

5 (RWD or RWE).ti,ab,kf. 1172

6 ((nonrandom* or non-random* or nonRCT or non-RCT or unrandom* or un-random* or nRCT) adj3 (evidence or study or studies or trial or trials or data)).ti,ab,kf. 26518

7 ((non-comparative or noncomparative) adj3 (evidence or study or studies or trial or trials or data)).ti,ab,kf. 2743

8 real world.ti,ab,kf. 79277

9 (observational adj3 (evidence or study or studies or trial or trials or data)).ti,ab,kf. 223776

10 or/1-9 489057

11 exp drug therapy/ 1508498

12 dt.fs. 2606804

13 (drug? or medication? or medicament? or medicine? or pharmaceutical?).ti,ab,kf. 2972045

14 or/11-13 5449549

15 (systematic review or meta-analysis).pt. 316130

16 meta-analysis/ or systematic review/ or systematic reviews as topic/ or meta-analysis as topic/ or exp technology assessment, biomedical/ or network meta-analysis/ 354475

17 ((systematic* adj3 (review* or overview*)) or (methodologic* adj3 (review* or overview*))).ti,ab,kf. 322529

18 ((quantitative adj3 (review* or overview* or synthes*)) or (research adj3 (integrati* or overview*))).ti,ab,kf. 15727

19 ((integrative adj3 (review* or overview*)) or (collaborative adj3 (review* or overview*)) or (pool* adj3 analy*)).ti,ab,kf. 39028

20 (data synthes* or data extraction* or data abstraction*).ti,ab,kf. 40684

21 (handsearch* or hand search*).ti,ab,kf. 11163

22 (mantel haenszel or peto or der simonian or dersimonian or fixed effect* or latin square*).ti,ab,kf. 35809

23 (met analy* or metanaly* or technology assessment* or HTA or HTAs or technology overview* or technology appraisal*).ti,ab,kf. 12193

24 (meta regression* or metaregression*).ti,ab,kf. 14630

25 (meta-analy* or metaanaly* or systematic review* or biomedical technology assessment* or bio-medical technology assessment*).mp,hw. 469803

26 (medline or cochrane or pubmed or medlars or embase or cinahl).ti,ab,hw. 343588

27 (cochrane or (health adj2 technology assessment) or evidence report).jw. 21481

28 (comparative adj3 (efficacy or effectiveness)).ti,ab,kf. 17614

29 (outcomes research or relative effectiveness).ti,ab,kf. 11253

30 ((indirect or indirect treatment or mixed-treatment or bayesian) adj3 comparison*).ti,ab,kf. 4342

31 (multi* adj3 treatment adj3 comparison*).ti,ab,kf. 295

32 (mixed adj3 treatment adj3 (meta-analy* or metaanaly*)).ti,ab,kf. 179

33 umbrella review*.ti,ab,kf. 1512

34 (multi* adj2 paramet* adj2 evidence adj2 synthesis).ti,ab,kf. 14

35 (multiparamet* adj2 evidence adj2 synthesis).ti,ab,kf. 18

36 (multi-paramet* adj2 evidence adj2 synthesis).ti,ab,kf. 12

37 or/15-36 686707

38 10 and 14 and 37 18003

39 exp animals/ not humans/ 5137964

40 (news or editorial or case reports).pt. or case report.ti. 3267159

41 or/39-40 8340564

42 38 not 41 17568

43 limit 42 to english language 17276

44 limit 43 to yr="2023" 1194

Key to Ovid symbols and commands:

* Unlimited right-hand truncation symbol

*N Limited right-hand truncation - restricts the number of characters following the word to N

ti,ab,kf,rn,nm,ot. Searches are restricted to the Title (ti), Abstract (ab), Keyword Heading Word (kf), Registry Number/Name of Substance (rn), Name of Substance Word (nm) and Original Title (ot) fields

adjN Retrieves records that contain terms (in any order) within a specified number (N) of words of each other

/ Searches are restricted to the Subject Heading field

exp The subject heading is exploded

pt. Search is restricted to the publication type field

or/1-20 Combines sets 1 to 20 using OR

Saved in Ovid as: temp-biogen-sma-protocol-med-021222

Final search used - single arm - methods project - CDSR

Search date: 17 July 2023

Cochrane Database of Systematic Reviews

Issue 7 of 12, July 2023

#1 "single arm":ti,ab,kw 4303

#2 (chart next review?):ti,ab,kw 2434

#3 "case series":ti,ab,kw 3395

#4 (uncontrolled near/3 (evidence or study or studies or trial or trials or data)):ti,ab,kw 1790

#5 (RWD or RWE):ti,ab,kw 208

#6 ((nonrandom* or non next random* or nonRCT or non next RCT or unrandom* or un next random* or nRCT) near/3 (evidence or study or studies or trial or trials or data)):ti,ab,kw 6510

#7 ((non next comparative or noncomparative) near/3 (evidence or study or studies or trial or trials or data)):ti,ab,kw 567

#8 "real world":ti,ab,kw 8015

#9 (observational near/3 (evidence or study or studies or trial or trials or data)):ti,ab,kw 18339

#10 #1 or #2 or #3 or #4 or #5 or #6 or #7 or #8 or #9 43068

#11 [mh "drug therapy"] 180455

#12 MeSH descriptor: [] explode all trees and with qualifier(s): [drug therapy - DT] 264180

#13 (drug? or medication? or medicament? or medicine? or pharmaceutical?):ti,ab,kw 835588

#14 #11 or #12 or #13 854253

#15 #10 and #14 with Cochrane Library publication date Between Jan 2023 and Dec 2023, in Cochrane Reviews, Cochrane Protocols 10

## Study Selection

Record assessment will be undertaken by one reviewer with a check of all included systematic reviews by a second reviewer.

Where primary studies on a particular subject are reported in more than one systematic review, the most recent and/or comprehensive review will be used and the other review will not be included.

## Data Extraction

We will develop a data extraction sheet in Excel and pilot it on a number of studies before progressing to full data extraction. One researcher will extract data from the eligible studies.

We will extract data related to the following research questions:

- What type of studies are included in reviews of non-comparative studies?
- What risk of bias tools are used for the assessment of single-arm cohort studies and single-arm trials?
- Is there a systematic difference in outcome for different study designs?

We will extract the following elements for the single-arm studies in the included systematic reviews:

- Study details (bibliographic details).
- Characteristics of included studies:
  - Count of the number of included primary studies of each study design.
- Details of intervention:
  - Primary treatment evaluated or intervention with the most contributing primary studies.
- Quality assessment tool used for each type of study design.
- If the SR also conducted a meta-analysis, we will extract outcome data:
  - for the primary outcome (or outcome with the largest number of contributing studies if no primary outcome stated)
  - for the intervention with the most contributing studies (if there is more than one intervention in the review)

## Synthesis and Analysis

If there is sufficient data, we will conduct analyses (sub-group analyses using e.g. OpenMeta [Analyst]) to investigate:

- Is there a difference in outcome for different study designs?

R:\General\Useful Resources\Systematic Review Folder of Greatness\1 Methods - investigations\Working groups projects\Study design\Single arm studies\Single-arm studies Protocol v1.0 22.09.2023

# Appendix A – Protocol Amendments

The protocol was agreed by email on [date].

The following protocol amendments (PA) were made subsequent to agreement of the final protocol:

- [Summary of amendment].

| Date of amendment | Protocol Section | PA number | Description of change |
| --- | --- | --- | --- |
| 22/09/23 | 2.3 | 1 | Exclusion of safety only reviews |
| 05/11/24 | Throughout | 2 | Change of reference to ‘case series’ to ‘single-arm cohort studies’ |
|  |  |  |  |
|  |  |  |  |
|  |  |  |  |
|  |  |  |  |
|  |  |  |  |
|  |  |  |  |
|  |  |  |  |
|  |  |  |  |

PA: Protocol Amendment
