# Supplementary material for: Single‐arm interventional versus observational studies for assessing efficacy: A meta‐epidemiological study
Source: Cochrane Evid Synth Methods. 2025 Jan 16;3(1):e70016. doi: 10.1002/cesm.70016 (PMC11795979; doi:10.1002/cesm.70016)
Supplement: Supplementary file 2 — Supporting information. [file CESM-3-e70016-s001.docx]

PRISMA checklist for epidemiological research

| **Section/topic** | **Proposed item to be used in methodology research** | **Location where item is reported** |
| --- | --- | --- |
| **Title** | |  |
| Title | Identify the report as a meta-epidemiologic study. | Title page |
| **Abstract** | |  |
| Structured summary | Provide a structured summary that includes the background of the topic, goal of the study, data sources, method of data selection, appraisal and synthesis methods, results, limitations, conclusions and implications of key findings. | Abstract |
| **Introduction** | |  |
| Rationale | Describe the rationale for the meta-epidemiological study in the context of what is already known. | Page 3 |
| Objectives | Provide an explicit statement of the goal of the meta-epidemiological study and the hypothesis being empirically tested. | Page 3 |
| **Methods** | |  |
| Protocol | Indicate if a protocol exists, if and where it can be accessed (eg, Web address). Registration of a protocol is not mandatory. | Page 3 |
| Eligibility criteria | Specify study characteristics used as criteria for eligibility with a rationale. | Page 4 |
| Information sources | Describe all information sources (eg, databases with dates of coverage, contact with experts to identify additional studies, Internet searches) and search date. | Page 4 |
| Search | Present full electronic search strategy for at least one database, including any limits used, such that it could be repeated. Search is commonly not driven by a clinical question. | Supplementary material 1 (Protocol) |
| Study selection | Describe the process for selecting studies for inclusion (ie, how many reviewers selected studies, reviewing in duplicate or by single individuals). | Page 4 |
| Data collection process | Describe method of data extraction from reports (eg, piloted forms, independently, in duplicate) and any processes used for manipulating data or obtaining and confirming data from investigators. | Page 4 |
| Data items | List and define all variables for which data were sought and any assumptions and imputations made. | Page 4-5 |
| Risk of bias in individual studies | If risk of bias assessment of individual studies was relevant to the analysis, describe the items used and how this information is to be used during data synthesis. | Not completed |
| Summary measures | State the principal summary measures (eg, ratio of risk ratios, difference in means) and explain its meaning and direction to readers. | Page 5 |
| Synthesis of results | Describe the statistical or descriptive methods of synthesis including measures of consistency if relevant. If applicable, describe the development of statistical or simulation modelling based on theoretical background. Describe and justify assumptions and computational approximations. Describe methods of additional analyses (eg, sensitivity or subgroup analyses, meta-regression), if done, indicating which were prespecified. | Page 5 |
| **Results** | |  |
| Study selection | Give numbers of studies assessed for eligibility and included in the study, with reasons for exclusions at each stage, ideally with a flow diagram. Present a measure of inter-reviewer agreement (eg, kappa statistic). | Page 5 |
| Study characteristics | For each study, present characteristics for which data were extracted and provide the citations. Clinical characteristics may not always be relevant. | Page 6-7 |
| Risk of bias within studies | If risk of bias assessment of individual studies was used in the meta-epidemiological analysis, report risk of bias indicators of each study to allow replication of findings. | NA |
| Results of individual studies | Present data elements used in the meta-epidemiological analysis from each study (results of clinical outcomes may not be relevant). | Figure 1 |
| Synthesis of results | Present results of statistical analysis done, including measures of precision and measures of consistency. Present validity of assumptions and fit of statistical or simulation modelling, if applicable. | Figure 2, page 8 |
| Additional analysis | Give results of additional analyses, if done (eg, sensitivity or subgroup analyses, meta-regression). | Figure 3, page 8 |
| **Discussion** | |  |
| Summary of evidence | Summarise the main findings and compare them with existing knowledge about the topic. The quality of evidence may not be relevant; however, investigators should describe their certainty in the results to readers. | Page 9-10 |
| Limitations | Discuss limitations at research methodology level (eg, likelihood of reporting or publication bias). | Page 11 |
| Conclusions | Provide general interpretation of the results and implications for future research. Provide any plausible impact on clinical practice. | Page 12 |
| **Funding** | |  |
| Funding | Describe sources of funding for the methodology research and role of funders. | Page 12 |
